# SCF^βTrCP^-mediated degradation of SHARP1 in triple-negative breast cancer

**DOI:** 10.1038/s41419-023-06253-6

**Published:** 2023-11-08

**Authors:** Juliana Haydeé Enriqué Steinberg, Fabiana Alejandra Rossi, Roberto Magliozzi, Laurensia Yuniati, Matteo Santucci, Mario Rossi, Daniele Guardavaccaro, Angela Lauriola

**Affiliations:** 1 Department of Biotechnology, University of Verona, Strada Le Grazie 15, 37134 Verona, Italy; 2 Instituto de Investigaciones en Medicina Traslacional (IIMT), CONICET-Universidad Austral, Av. Juan Domingo Perón 1500, B1629AHJ Pilar, Argentina; 3 Facultad de Ciencias Biomédicas, Universidad Austral, Av. Juan Domingo Perón 1500, B1629AHJ Pilar, Argentina; 4 Hubrecht Institute-KNAW and University Medical Center Utrecht, Uppsalalaan 8, 3584 CT Utrecht, The Netherlands

**Keywords:** Ubiquitin ligases, Cancer

## Abstract

Triple-negative breast cancer (TNBC) is a subtype of breast cancer associated with metastasis, high recurrence rate, and poor survival. The basic helix-loop-helix transcription factor SHARP1 (Split and Hairy-related Protein 1) has been identified as a suppressor of the metastatic behavior of TNBC. SHARP1 blocks the invasive phenotype of TNBC by inhibiting hypoxia-inducible factors and its loss correlates with poor survival of breast cancer patients. Here, we show that SHARP1 is an unstable protein that is targeted for proteasomal degradation by the E3 ubiquitin ligase complex SCF^βTrCP^. SHARP1 recruits βTrCP via a phosphodegron encompassing Ser240 and Glu245 which are required for SHARP1 ubiquitylation and degradation. Furthermore, mice injected with TNBC cells expressing the non-degradable SHARP1(S240A/E245A) mutant display reduced tumor growth and increased tumor-free survival. Our study suggests that targeting the βTrCP-dependent degradation of SHARP1 represents a therapeutic strategy in TNBC.

## Introduction

Triple-negative breast cancer (TNBC) is a highly heterogeneous breast cancer subtype that accounts for ~15% of all breast cancers [1]. As TNBC cells do not express estrogen and progesterone hormone receptors (ER and PR) and epidermal growth factor receptor 2 (HER2), it is difficult to develop a successful therapeutic strategy for TNBC. Recently, a basic helix-loop-helix transcription factor, named SHARP1 (Split and Hairy-related Protein 1) has been identified as a suppressor of the invasive and metastatic phenotype of TNBC. SHARP1 has been shown to promote proteasomal degradation of the hypoxia-inducible factors HIF-1α and HIF-2α, thereby blocking the metastatic behavior of TNBC [2, 3]. Furthermore, loss of SHARP1 expression correlates with poor survival of breast cancer patients and represents a prognostic marker for TNBC. Other studies have demonstrated that SHARP1 is induced in the bone marrow by TGFβ2 signaling and mediates the dormancy of malignant dissemination of tumor cells by controlling the expression of the CDK inhibitor p27 [4]. Interestingly, SHARP1 is known to inhibit the CLOCK/BMAL1-mediated transactivation of the core circadian clock component PER1 by interacting with BMAL1 or competing for E-box binding sites in the promoter of PER1 [5]. SHARP1 regulates sleep length in mammals. Indeed, a single SHARP1 mutation (P385R) is associated with a short sleep phenotype in humans, and transgenic mice carrying the corresponding mutation display less sleep time and increased vigilance time when compared with control animals [6]. These two reported functions of SHARP1 as a suppressor of breast cancer metastasis and regulator of circadian rhythms and sleep length are intriguing because epidemiologic studies have demonstrated an increased breast cancer risk in long-term night shift workers [7]. Recently, it has been revealed that SHARP1 has oncogenic properties in MLL-AF6 Acute Myeloid Leukemia (AML), which has the worst prognosis among all subtypes of MLL-rearranged AMLs [8], indicating that SHARP1 can act either as an oncoprotein or a tumor suppressor depending on the cancer type.

Here, we show that SHARP1 is targeted for proteasomal degradation by the SCF^βTrCP1^ ubiquitin ligase complex in TNBC cells and that inhibition of SHARP1 degradation may suppress TNBC in vivo.

## Results

### SHARP1 binds the SCF^βTrCP^ ubiquitin ligase complex

**Fig. 1:**
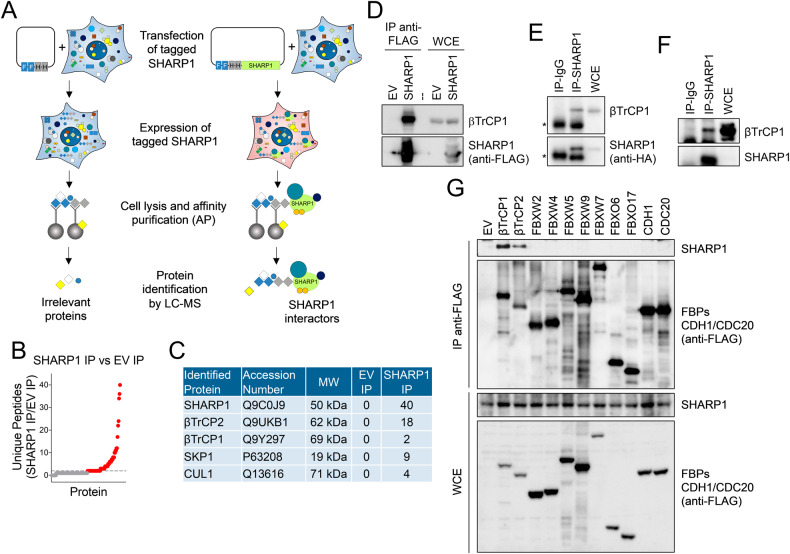
SHARP1 interacts with βTrCP1 and βTrCP2. **A** Overview of the experimental workflow aimed at the identification of SHARP1 interactors. FLAG-HA-tagged SHARP1 was expressed in HEK293T cells. Cells were lysed and whole-cell extracts were subjected to immunoprecipitation using FLAG and HA beads. SHARP1 immunoprecipitates were then analyzed by mass spectrometry. **B** Dot plot showing the number of unique peptides detected by mass spectrometry after SHARP1 immunoprecipitation (IP) compared to the control immunoprecipitation (EV: empty vector). **C** The number of unique peptides, accession numbers, and molecular weights (MW) for selected proteins recovered by mass spectrometry in control (EV: empty vector) and SHARP1 immunopurifications (IP) are shown. **D** HEK293T cells were transfected with an empty vector (EV) or FLAG-tagged SHARP1. Whole-cell extracts (WCE) were immunoprecipitated (IP) with anti-FLAG resin and immunocomplexes were probed with the indicated antibodies. **E**, **F** Whole-cell extracts (WCE) from MDA-MB231 cells stably expressing HA-tagged SHARP1 **E** or parental MDA-MB231 cells **F** were immunoprecipitated with an anti-SHARP1 antibody. SHARP1 immunocomplexes were probed with antibodies to the indicated proteins. **G** HEK293T cells, transfected with the indicated FLAG-tagged F-box proteins and the APC/C subunits CDH1 and CDC20, were lysed. WCEs were immunoprecipitated (IP) with anti-FLAG resin and analyzed by immunoblotting with antibodies for the indicated proteins.

To identify SHARP1 interactors, FLAG-HA-epitope tagged SHARP1 was expressed in HEK293T cells and immunopurified (Fig. 1A). Mass spectrometry analysis of affinity-purified SHARP1 identified peptides corresponding to the F-box proteins βTrCP1 and βTrCP2 as well as the SCF core components SKP1 and CUL1 (Fig. 1B, C). βTrCP1 and βTrCP2 are two biochemically indistinguishable βTrCP paralogs expressed in mammalian cells, hence, we will utilize the protein symbol βTrCP when pointing out both. The binding of SHARP1 to βTrCP1 was confirmed by immunoprecipitation followed by immunoblotting in both HEK293T cells and MDA-MB231 breast cancer cells (Fig. 1D–F and Supplementary File 1). To assess the specificity of the interaction of SHARP1 with βTrCP1 and βTrCP2, we immunoprecipitated nine FLAG-tagged F-box proteins as well as the APC/C subunits CDC20 and CDH1 from HEK293T cells. Immunoblotting analysis showed that only βTrCP1 and βTrCP2 coimmunoprecipitated with endogenous SHARP1 (Fig. 1G and Supplementary File 1).

### SHARP1 abundance is controlled by SCF^βTrCP^

**Fig. 2:**
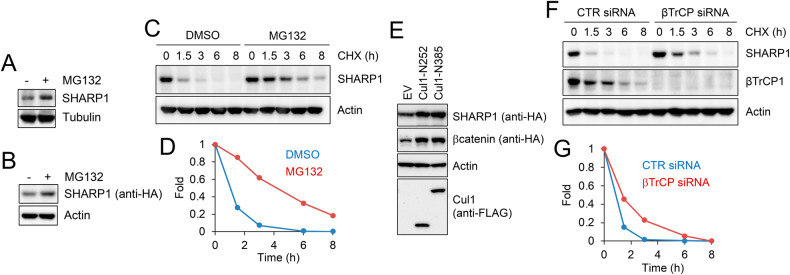
Characterization of SHARP1 degradation. **A**, **B** Parental MDA-MB231 cells **A** or MDA-MB231 cells stably expressing HA-tagged SHARP1 **B** were treated with the proteasome inhibitor MG132 for 6 hours. Cells were lysed and whole-cell extracts were subjected to immunoblotting with antibodies for the indicated proteins. **C** MDA-MB231 cells were treated with cycloheximide (CHX) with or without the proteasome inhibitor MG132, collected at the indicated times, and lysed. Whole-cell extracts were subjected to immunoblotting with the indicated antibodies. **D** The graph represents the quantification of SHARP1 levels (shown in C) normalized to the Actin loading control. **E** Cells were transfected with an empty vector (EV) or FLAG-tagged CUL1 dominant-negative mutants (CUL1-N252 or CUL1-N385) along with HA-tagged SHARP1 or HA-tagged β-catenin. Cells were collected and lysed. Whole-cell extracts were analyzed by immunoblotting with the indicated antibodies. **F** Cells were transfected with a siRNA targeting both βTrCP1 and βTrCP2 or a control siRNA and treated with cycloheximide (CHX) for the indicated times. Cells were lysed and whole-cell extracts were analyzed by immunoblotting with the indicated antibodies. **G** The graph represents the quantification of SHARP1 levels (shown in **F**) normalized to the Actin loading control.

The interaction of SHARP1 with an SCF ubiquitin ligase complex suggested that SHARP1 abundance might be regulated by the proteasome. To test this hypothesis, MDA-MB231 breast cancer cells (parental or stably expressing HA-tagged SHARP1) were treated with the proteasome inhibitor MG132, and SHARP1 levels were examined by immunoblotting. As shown in Fig. 2A, B, and Supplementary File 1, MG132 treatment resulted in SHARP1 accumulation. Accordingly, when we treated cells with cycloheximide (an inhibitor of protein synthesis), we observed a decrease in SHARP1 levels which was prevented by MG132 treatment (Fig. 2C, D and Supplementary File 1). Moreover, ectopic expression of dominant-negative CUL1 mutants or silencing of βTrCP expression by RNAi resulted in SHARP1 accumulation in cells (Fig. 2E–G and Supplementary File 1). Altogether these results indicate that SHARP1 is degraded via an SCF^βTrCP^- and proteasome-dependent mechanism.

### Ser240 and Glu245 in SHARP1 are required for its binding to βTrCP1

**Fig. 3:**
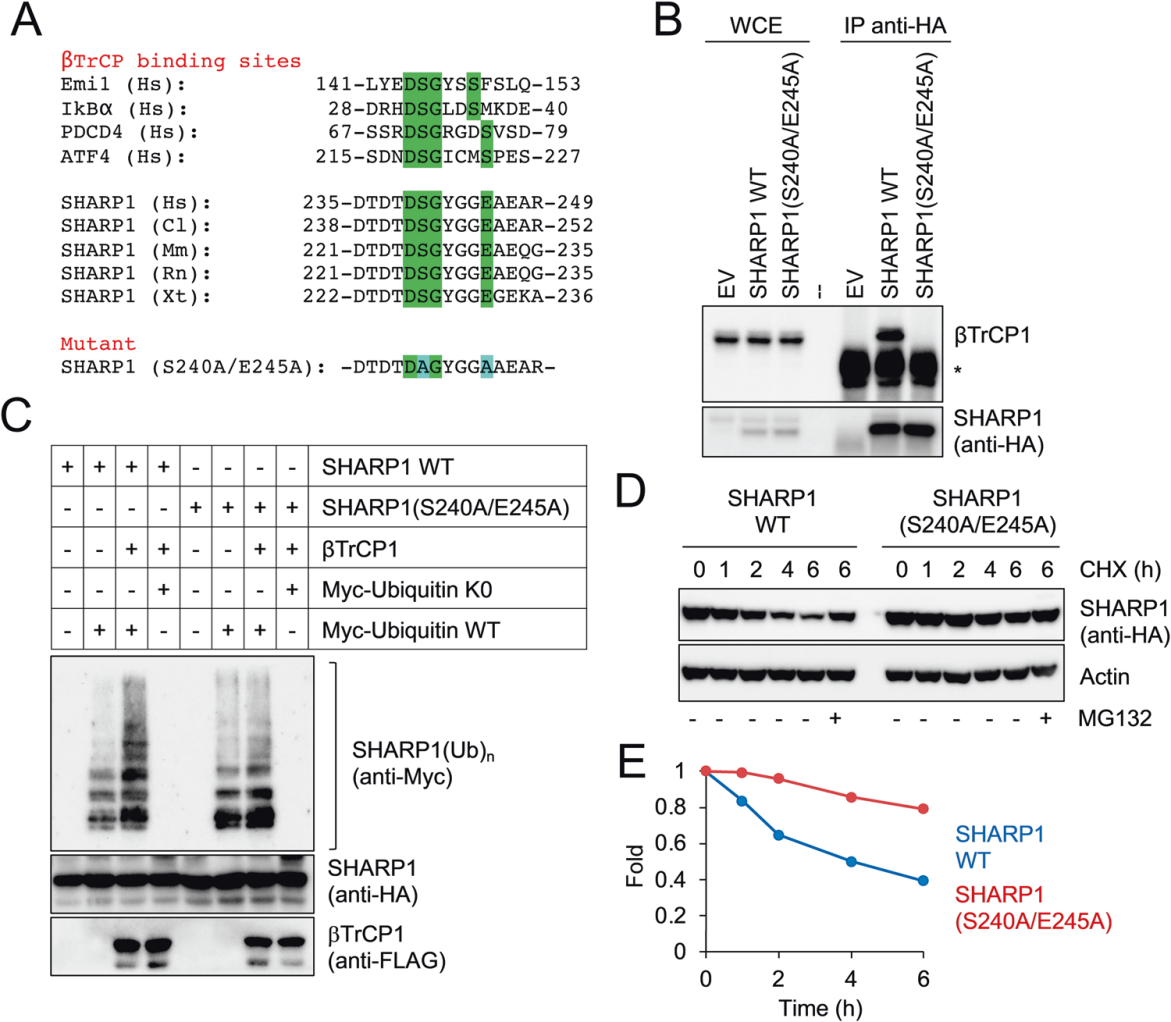
Ser240 and Glu245 in SHARP1 are required for SHARP1-βTrCP1 interaction, SHARP1 ubiquitylation, and proteasomal degradation. **A** Alignment of the amino acid regions encompassing the βTrCP-binding motif (highlighted in green) in previously reported βTrCP substrates and SHARP1 orthologs. The amino acid sequence of the SHARP1 phosphodegron mutant is shown at the bottom, with the altered amino acids highlighted in blue. Hs, Homo sapiens; Cl, Canis lupus familiaris; Mm, Mus musculus; Rn, Rattus norvegicus; Xt, Xenopus tropicalis. **B** MDA-MB231 parental cells or expressing HA-tagged wild-type SHARP1 or the SHARP1(S240A/E245A) mutant were lysed. Whole-cell extracts were subjected to immunoblotting with HA resin, followed by immunoblotting. Asterisk indicates the immunoglobulin heavy chain. **C** HEK293T cells were transfected with HA-tagged SHARP1 (wild type or S240A/E245A) and MYC-tagged ubiquitin [wild type or lysine-less (K0)], with or without FLAG-tagged βTrCP1. Cells were treated with the proteasome inhibitor MG132 for 5 hours and lysed. Whole-cell extracts were prepared in denaturing conditions and immunoprecipitated with an anti-HA antibody. Immunoprecipitates were then immunoblotted with the indicated antibodies. **D** MDA-MB231 cells expressing wild-type (WT) SHARP1 or the SHARP1(S240A/E245A) mutant were treated with cycloheximide (CHX). Cells were collected at the indicated times and lysed. Whole-cell extracts were subjected to immunoblotting with antibodies specific to the indicated proteins. **E** The graph represents the quantification of SHARP1 levels shown in **D** normalized to the Actin loading control.

Analysis of SHARP1 amino acid sequence revealed the presence of a putative βTrCP-binding domain resembling a phosphodegron characteristic of SCF^βTrCP^ substrates (Fig. 3A). The reported consensus sequence for this motif is DpSGXX(X)pS, in which either of the phosphorylated serine residues can be replaced by aspartic or glutamic acid [9–11]. The potential βTrCP-binding region in SHARP1 is evolutionarily conserved and encompasses Ser240 and Glu245. To assess whether Ser240 and Glu245 are required for the interaction of SHARP1 with βTrCP1, we expressed in MDA-MB231 cells HA-epitope tagged wild type SHARP1 and the SHARP1(S240A/E245A) mutant which were then immunoprecipitated. As shown in Fig. 3B and Supplementary File 1, wild-type SHARP1, but not SHARP1(S240A/E245A), coimmunoprecipitated with endogenous βTrCP1.

### Ser240 and Glu245 in SHARP1 are required for their ubiquitylation and degradation

We then examined if βTrCP expression is able to promote SHARP1 polyubiquitylation. HA-tagged SHARP1 (wild type or degron mutant) was expressed in cells along with MYC-tagged ubiquitin (wild type or Lysine-less mutant) with or without FLAG-tagged βTrCP1. Cells were then lysed and SHARP1 was immunoprecipitated in denaturing conditions. Immunoblotting analysis using an anti-MYC antibody to detect ubiquitylated proteins showed that βTrCP1 stimulated polyubiquitylation of wild-type SHARP1 but not the one of the SHARP1(S240A/E245A) mutant (Fig. 3C and Supplementary File 1).

Next, we tested whether SHARP1 Ser240 and Glu245 are required for its proteasome-dependent degradation. HA-epitope tagged wild-type SHARP1 and SHARP1(S240A/E245A) were expressed in MDA-MB231 breast cancer cells, which were then treated with cycloheximide. Immunoblotting analysis showed that compared with wild-type SHARP1, the half-life of the SHARP1(S240A/E245A) mutant was increased in breast cancer cells (Fig. 3D, E and Supplementary File 1).

### SHARP1 degradation controls migration of TNBC cells in vitro and TNBC growth in vivo

**Fig. 4:**
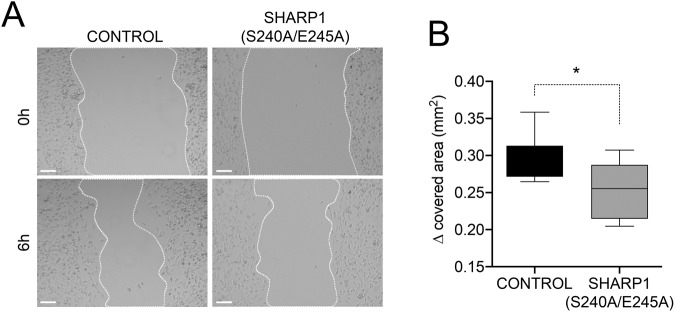
SHARP1 degradation controls migration of triple-negative breast cancer cells. **A** MDA-MB231 control cells or expressing the SHARP1(S240A/E245A) mutant were grown to confluence. Cell monolayers were wounded with a micropipette tip and photographed immediately after wounding (0 h) and after 6 hours (6 h). Representative areas at the indicated time points are shown. Scale bar = 100 μm. **B** The graph represents the Δ covered area (mm^2^) after 6 h (*n* = 9, Student’s *t* test, *p* = 0.0121).

To assess the role of SHARP1 degradation in breast cancer cell migration, we employed the wound-closure assay, which monitors the migration of cells into a scratch made in a confluent monolayer of cells. As shown in Fig. 4A, B, MDA-MB231 cells expressing the non-degradable SHARP1(S240A/E245A) mutant displayed significantly reduced migration speed compared with control cells.

**Fig. 5:**
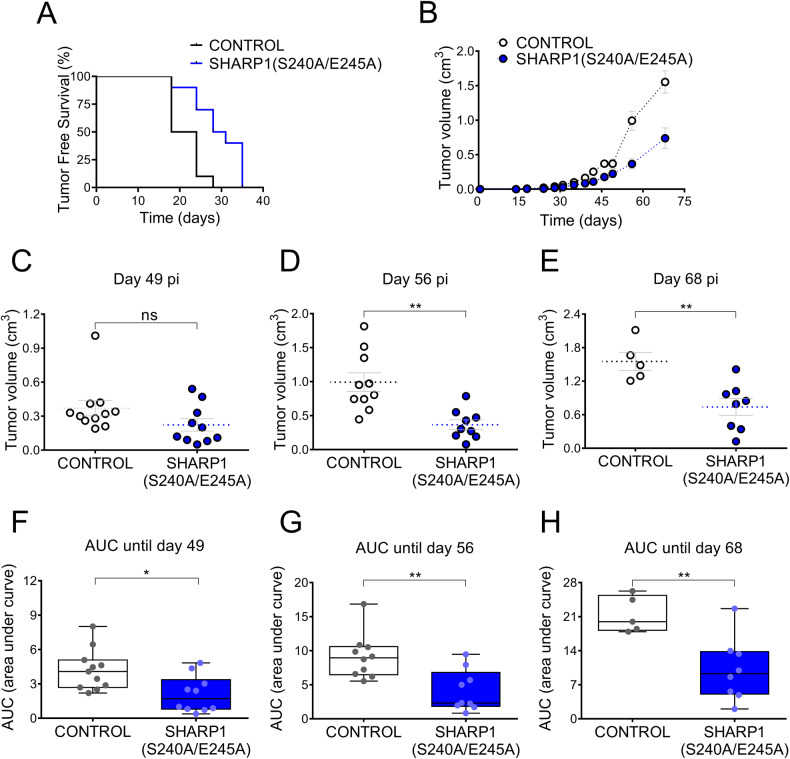
Expression of the SHARP1(S240A/E245A) mutant inhibits triple-negative breast cancer in vivo. **A** MDA-MB231 control cells or expressing the SHARP1(S240A/E245A) mutant were inoculated subcutaneously into the mammary fat pads of female NOD/SCID mice. Kaplan–Meier curves were built for Tumor-Free Survival (TFS) over time (*n* ≥ 10, Log-Rank (Mantel–Cox) test, *p* value = 0.0024). Hazard ratio (logrank): 2.625 [95% CI of ratio = 0.9845–6.997]. Number of mice used in each treatment: 11 control, 10 SHARP1(S240A/E245A) mutant. **B** Tumor volume was calculated at the indicated time points (mean value ± S.E.). **C**–**E** Tumor volume at the indicated time points post-injection (pi). Unpaired two-tailed *t* test. *P* values: 0.1104 (ns), 0.0012 (**), and 0.0045 (**) for tumor volume differences at days 49, 56, or 68 post-injection, respectively. **F**–**H** Area under the curve (AUC) until the indicated time points. Unpaired two-tailed *t* test. *P* values: 0.0100 (*), 0.0031 (**), and 0.0050 (**) for the area under curve differences (curves ending at day 49, 56, or 68 post-injection, respectively).

To study the effect of SHARP1 accumulation in vivo, MDA-MB231 cells expressing SHARP1(S240A/E245A) were injected subcutaneously into the mammary fat pad of female mice. As shown in Fig. 5A, tumor-free survival of mice injected with MDA-MB231 cells expressing the degradation-resistant SHARP1 mutant was longer when compared with one of mice injected with control cells. Moreover, tumor growth analysis indicated that tumors generated from SHARP1(S240A/E245A) cells were considerably less volumetric than the ones originating from control cells (Fig. 5B–H). Altogether, these results indicate that failure to degrade SHARP1 impairs TNBC in vivo.

## Discussion

In the present work, we have demonstrated that SHARP1 is targeted for proteasomal degradation by the SCF^βTrCP^ ubiquitin ligase complex. Further studies are required to understand the temporal and spatial regulation of SHARP1 destruction in the cell. The identification of the kinase responsible for the phosphorylation of the SHARP1 degron (Ser240), and possibly the priming kinases involved, would be crucial to reveal the regulation of SHARP1 polyubiquitylation and degradation.

It has been reported that SHARP1 is a key suppressor of the invasive and metastatic phenotype in triple-negative breast cancer [2]. Hence, βTrCP-dependent degradation of SHARP1 points to an oncogenic role of βTrCP in breast cancer. This is in agreement with previous studies demonstrating that βTrCP overexpression, which is frequent in human epithelial cancers, results in oncogenic transformation of human mammary epithelial cells [12]. Moreover, by employing an shRNA-based functional selection screen aimed at the identification of ubiquitylation pathway genes that positively regulate cell migration in breast cancer cells, we found that βTrCP knockdown decreases the migratory and invasive potential of triple-negative breast cancer cells [13] further supporting an oncogenic function of βTrCP in breast cancer.

We have shown that failure to degrade SHARP1 impairs triple-negative breast cancer suggesting that pharmacological strategies aimed at blocking the interaction of SHARP1 with βTrCP would represent a potential anticancer therapeutic approach. Indeed, small-molecule compounds that disrupt the physical association between SHARP1 and βTrCP would result in SHARP1 stabilization providing beneficial effects against the metastatic spread of breast cancer cells. In this regard, previous studies demonstrated the feasibility of blocking E3-substrate interactions by competitive small-molecule inhibitors [14]. These strategies have been successful, especially for E3 substrate-receptor subunits (e.g., CDC20, FBXW7), which, as in the case of βTrCP, contain WD40 repeats as protein–protein interaction domain that mediates substrate binding. Other studies have identified allosteric inhibitors which embed themselves into deep pockets on the lateral surface of the WD40 β-propeller thus causing conformational changes that are propagated to the substrate-binding site [15]. In conclusion, our results suggest that targeting SHARP1 degradation represents a new therapeutic strategy in breast cancer patients.

## Methods

### Cell culture, transfection, and drug treatment

HEK293T, GP2-293, and MDA-MB231 cells were maintained in Dulbecco’s modified Eagle’s medium (DMEM; Thermo Fisher Scientific) containing 10% fetal bovine serum, 100 U/ml penicillin, and 100 μg/ml streptomycin. Cells were transfected by the polyethylenimine (PEI) method. A siRNA oligonucleotide (5′-GUGGAAUUUGUGGAACAU-3′) (Dharmacon, Boulder, CO, US) [16] targeting both human βTrCP1 (515–535) and βTrCP2 (262–282) was transfected into cells using Lipofectamine RNAiMAX (Thermo Fisher Scientific, Carlsbad, CA) according to manufacturer’s protocol. The following drugs were used: Z-Leu-Leu-Leu-H (MG132) (Peptide Institute, Osaka, Japan; 10 μM), cycloheximide (Merck, St Louis, MO 100 μg/ml).

### Biochemical methods

For preparation of cell extracts, cells were washed and collected in ice-cold PBS and lysed in Triton Lysis Buffer (TLB) (50 mM Tris pH 7.5, 250 mM NaCl, 0.1% Triton X-100, 1 mM EDTA, 50 mM NaF and protease and phosphatase inhibitors) for 30 minutes on ice, followed by centrifugation at 4 °C for 20 minutes. Cell extracts were subjected to either immunoblotting or immunoprecipitation followed by immunoblotting [17]. For immunoprecipitation, cells from one 15-cm dish were lysed in TLB as described above. Cell extracts were precleared by incubation with protein G- or protein A-Sepharose beads (Life Technologies, Rockford, USA) for 1 hour at 4 °C and then incubated with the indicated antibody for 3 hours at 4 °C. Protein G- or protein A-Sepharose beads were then added and incubated for 45 minutes. Beads were washed 4 times with TLB, and proteins were eluted in 5× Laemmli sample buffer. For immunoblotting, proteins were separated by SDS-polyacrylamide gel electrophoresis (SDS-PAGE), transferred onto a PVDF membrane (Millipore, Corck, Ireland), and incubated with the indicated antibodies.

To assess SHARP1 ubiquitylation in cultured cells, cells were transfected with pcDNA3-HA-SHARP1 (WT or S240A/E245A), pcDNA3-FLAG-βTrCP1, or pCW7-His-MYC-ubiquitin (WT or Lysine-less). After 48 hours, cells were treated with MG132 for 6 hours and harvested. Cells were lysed for 10 minutes at 95 °C in denaturing conditions (50 mM Tris pH 7.5, 150 mM NaCl, 1 mM EDTA, 1% SDS). After cooling, cell lysates were diluted 10 times using TLB (see above), sonicated, and subjected to immunoprecipitation using an anti-HA antibody. Ubiquitylated SHARP1 was detected by immunoblotting using an anti-MYC antibody.

Mouse monoclonal antibodies were from BD Biosciences (βCatenin #610153), Biolegend (HA #901514), Merck (FLAG, clone M2, #F3165, MYC #M5546), and Santa Cruz Biotechnology (Actin #sc-69879). Rabbit polyclonal antibodies were from Cell Signaling Technology (βTrCP1 # 4394 S, Wee1 #4936, HA #3724 S), Novus Biologicals (SHARP1 #NBP1-19613), Thermo Scientific (SHARP1 #MA537787) and Santa Cruz Biotechnology (Skp1 #sc-5281). Anti-mouse IgG (#NA931, Buckinghamshire, UK) and anti-rabbit IgG (#NA934, Buckinghamshire, UK) horseradish peroxidase (HRP)-conjugated secondary antibodies were from GE Healthcare. Affinity-purified rabbit IgGs were from Merck.

### Identification of SHARP1 interactors

HEK293T cells were transfected with pcDNA3-FLAG-HA-SHARP1 and treated with 10 μM MG132 for 5 hours. Cells were harvested and subsequently lysed in lysis buffer [50 mM Tris-HCl (pH 7.5), 150 mM NaCl, 1 mM EDTA, and 0.5% NP-40 plus protease and phosphatase inhibitors]. SHARP1 was immunopurified with anti-FLAG agarose resin (Merck, St Louis, MO). Beads were rinsed, and proteins were eluted by competition with FLAG peptide (Merck, St Louis, MO). The eluate was then subjected to a second immunopurification with anti-HA resin (12CA5 monoclonal antibody cross-linked to protein G–Sepharose; Invitrogen, Rockford, USA). Beads were rinsed, and proteins were eluted using RapiGest SF (Waters, Milford, MA, USA). Eluates were subsequently treated with reduction buffer (1 μg/μl dithiothreitol) for 30 minutes and alkylation buffer (5 μg/μl iodoacetamide) for 20 minutes, followed by Lys-C for 4 hours. Trypsin was then added at 1:50 ratio and the mixture was incubated overnight at 37 °C. Trypsin was quenched by adding trifluoroacetic acid (TFA). For MS analysis, peptides were first separated with a C18 column (Zorbax) and introduced by nanoelectrospray into the LTQ Orbitrap Elite (Thermo Fisher) and MS/MS in data-dependent decision tree mode (collision-induced dissociation/electron transfer dissociation) as described previously [18]. Raw files were converted to MGF files using Proteome Discoverer version 1.4 (Thermo Fisher). The non-fragment filter was used to simplify ETD spectra and the Top N filter (6 highest peaks admitted per 100 Da) for the CID spectra. All MGF files were submitted to Mascot search engine (version 3.0) via Proteome Discoverer version 1.4. Spectra were searched against the UniProt Human database (version 2013-07, 20,277 entries). Peptide tolerance was set to 15 ppm and MS/MS tolerance was set to 0.5 daltons. All peptide-spectrum matches (PSMs) were filtered at a Mascot score cutoff of 30. Only PSMs with a minimum length of 7 amino acids were kept.

### Plasmids, transient transfections, and lentivirus-mediated gene transfer

SHARP1, βTRCP1, βTRCP2, FBXW2, FBXW4, FBXW5, FBXW7, FBXW9, FBXO6, FBXO17, CDH1 and CDC20 cDNAs were cloned in pcDNA3.1. Wild type SHARP1 and the SHARP1(S240A/E245A) mutant were 2xFLAG-2xHA-tagged or single HA-tagged, whereas βTrCP1, FBXW2, FBXW4, FBXW5, FBXW7, FBXW9, FBXO6, FBXO17, CDH1 and CDC20 were FLAG-tagged. Wild-type SHARP1 and the SHARP1(S240A/E245A) mutant were subcloned into the pLXRN vector. The SHARP1(S204A/E245A) mutant was generated by site-directed mutagenesis (NEB Q5®/Strategene). All cDNAs were sequenced. Retroviruses were produced in GP2-293 cells by two-plasmid cotransfection. Cells were transfected with the pLXRN vector together with G protein of the vesicular stomatitis virus (VSV) vector encoding the envelope proteins. Supernatants were collected every 24 hours on two consecutive days starting 24 hours after transfection, filtered, and transferred to a 10-cm dish of MDA-MB231 cells in the presence of polybrene (4 μg/ml). After 16 hours, the medium was replaced with DMEM containing 10% fetal bovine serum, and neomycin for selection.

### Wound-closure assay

MDA-MB231 cells were seeded onto 24-well culture dishes at 2.5 × 10^5^ per well in growth medium. Confluent monolayers were starved for 24 hours in Dulbecco’s modified Eagle’s medium, containing 0.1% fetal bovine serum and a single scratch wound was made using a micropipette tip. Cells were washed with PBS to remove cell debris and incubated in DMEM containing 3% fetal bovine serum for 6 hours at 37 °C to enable cell migration into wounds. Images were acquired under bright field illumination using an EVOS™ FL digital inverted microscope. Nine independent experiments were performed consisting of at least three technical replicates each. Image analysis was performed by Fiji software, using a specific image J plugin for wound healing analysis [19].

### Mouse tumorigenesis

NOD SCID mice were originally purchased from Jackson Laboratories (Bar Harbor, ME, USA), and bred in the IBioBA’s animal facility under a pathogen-free environment. For all experiments, 7/8-week-old mice were used in accordance with protocols approved by the Institutional Board on Animal Research and Care Committee (CICUAL, Experimental Protocol # 63, 22 Nov 2016), Facultad de Ciencias Exactas y Naturales (School of Exact and Natural Sciences), University of Buenos Aires. Four weeks after birth, mice were randomly and equally divided into cages (no more than five animals/cage). At the time of injection, cages for the different treatments were arbitrarily selected. For in vivo tumor studies, 5 × 10^5^ transduced cells were suspended in 100 μl of sterile 1× PBS and subcutaneously injected into the mammary fat pads of female mice. Tumor volumes were calculated using the following formula: Vol (volume) = ½ (width^2^ × length). Area Under Curve analysis was performed using measurements from alive mice at each time point [13, 20, 21].

### Statistical analysis

All data represent the average from at least three independent experiments. For wound-closure assay: results are presented as box-and-whisker plots with median interquartile ranges plus minimum to maximum. *n* indicates the number of independent replicates. An unpaired Student’s *t* test was used to compare differences among samples. *P* value differences of <0.05 were considered statistically significant and GraphPad Prism (version 8.2.1) software was used.

For mouse tumorigenesis analysis: statistical analysis was performed using GraphPad Prism software (version 8.2.1, GraphPad Software, San Diego, CA, USA), applying a two-tailed Student’s *t* test. A *p* < 0.05 was considered statistically significant.

### Reporting summary

Further information on research design is available in the Nature Research Reporting Summary linked to this article.

### Supplementary information

Uncropped Western Blots

Reporting Summary

## Data Availability

The datasets generated during and/or analyzed during the current study are available from the corresponding author upon reasonable request.
