# Supplementary material for: SCFβTrCP-mediated degradation of SHARP1 in triple-negative breast cancer
Source: Cell Death Dis. 2023 Nov 8;14(11):726. doi: 10.1038/s41419-023-06253-6 (PMC10632515; doi:10.1038/s41419-023-06253-6)
Supplement: Supplementary file 1 — Uncropped Western Blots [file 41419_2023_6253_MOESM1_ESM.pptx]

## Slide 1
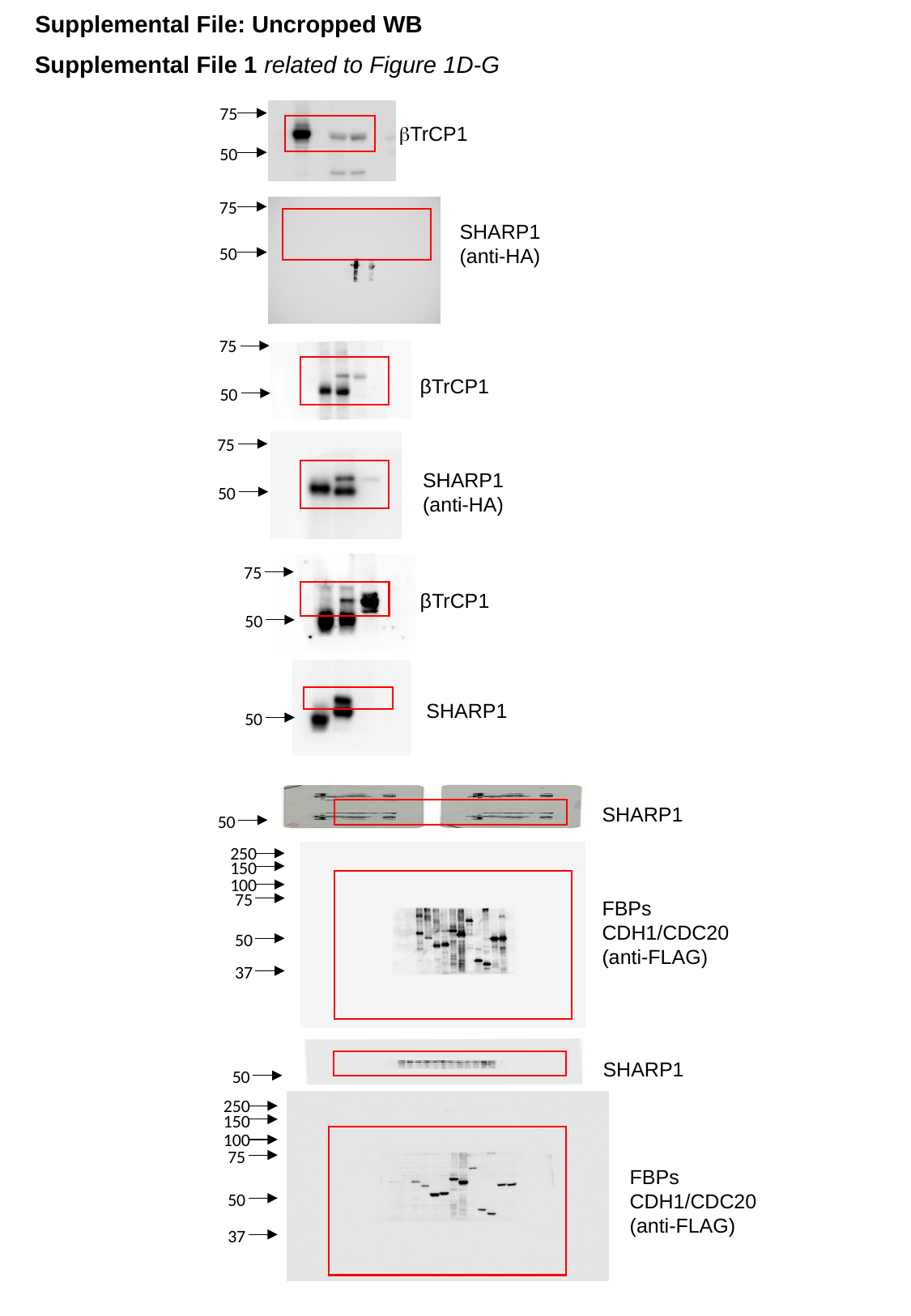

Supplemental File: Uncropped WB
Supplemental File 1 related to Figure 1D-G
75
bTrCP1
50
75
SHARP1
(anti-HA)
50
75
βTrCP1
50
75
SHARP1
(anti-HA)
50
75
βTrCP1
50
SHARP1
50
SHARP1
50
250
150
100
75
FBPs
CDH1/CDC20
(anti-FLAG)
50
37
SHARP1
50
250
150
100
75
FBPs
CDH1/CDC20
(anti-FLAG)
50
37

## Slide 2
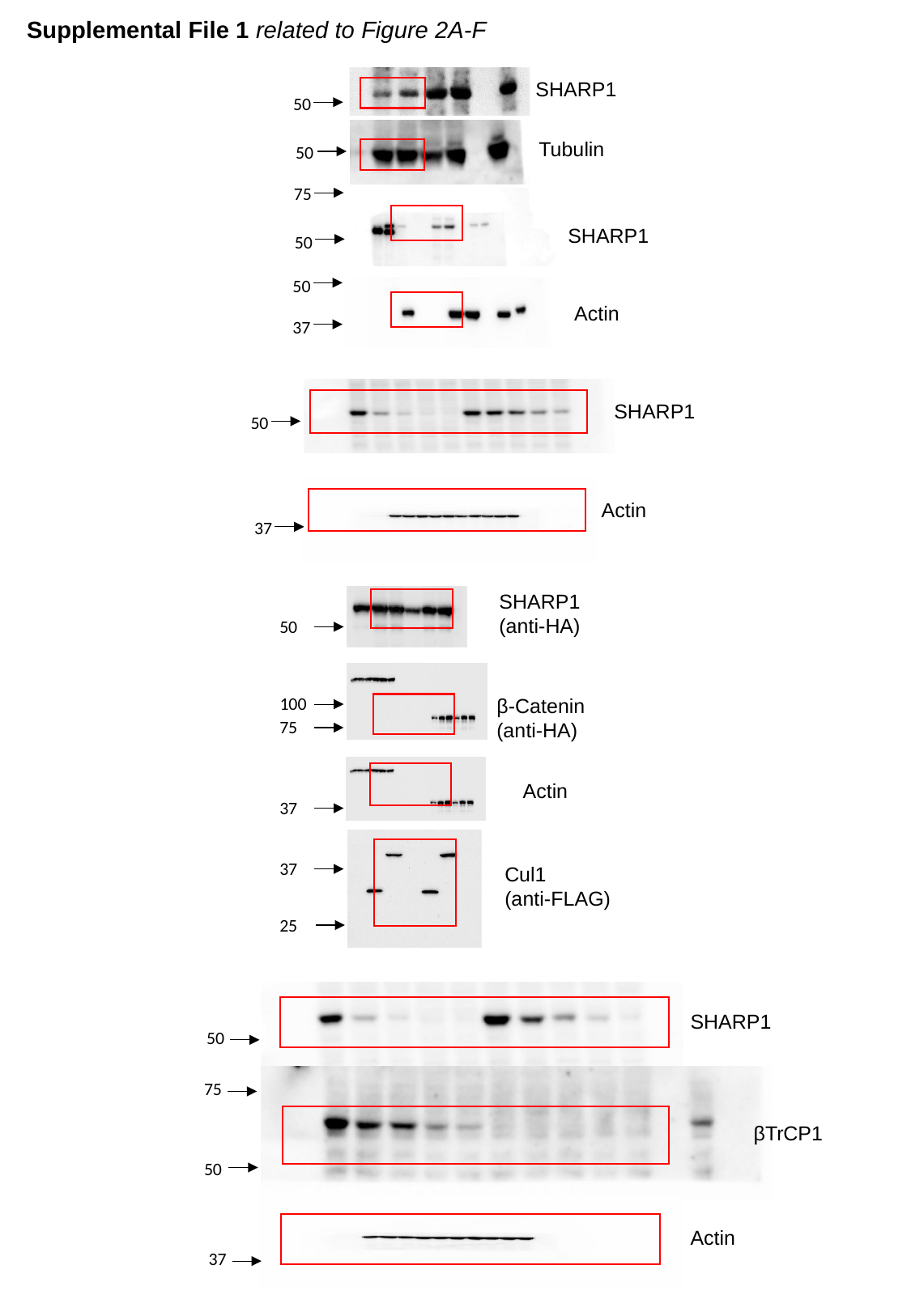

Supplemental File 1 related to Figure 2A-F
SHARP1
50
Tubulin
50
75
SHARP1
50
50
Actin
37
SHARP1
50
Actin
37
SHARP1
(anti-HA)
50
β-Catenin
(anti-HA)
100
75
Actin
37
37
Cul1
(anti-FLAG)
25
SHARP1
50
75
βTrCP1
50
Actin
37

## Slide 3
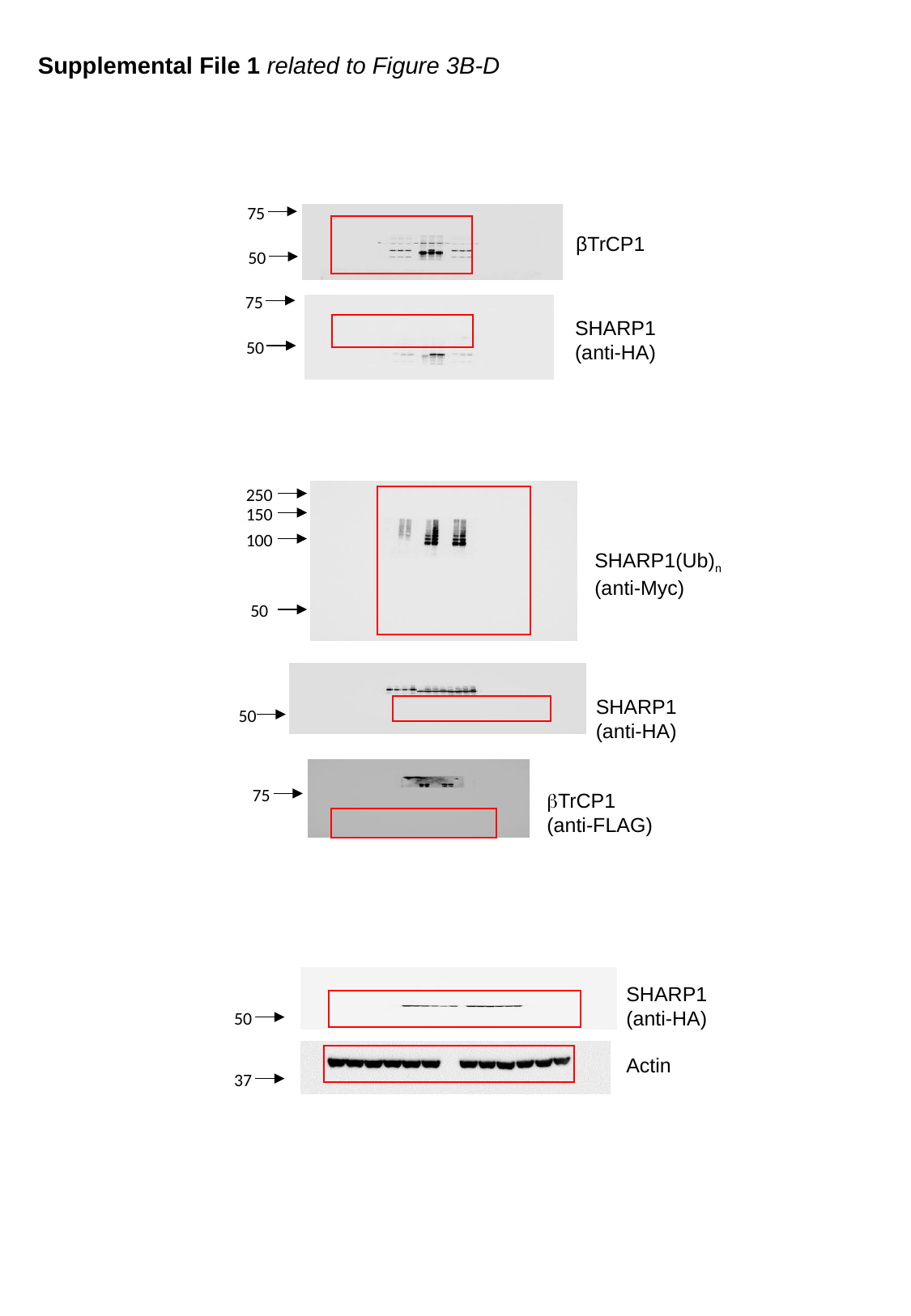

Supplemental File 1 related to Figure 3B-D
75
βTrCP1
50
75
SHARP1
(anti-HA)
50
250
150
100
SHARP1(Ub)n
(anti-Myc)
50
SHARP1
(anti-HA)
50
75
bTrCP1
(anti-FLAG)
SHARP1
(anti-HA)
50
Actin
37
